# Evolutionary age of repetitive element subfamilies and sensitivity of DNA methylation to airborne pollutants

**DOI:** 10.1186/1743-8977-10-28

**Published:** 2013-07-15

**Authors:** Hyang-Min Byun, Valeria Motta, Tommaso Panni, Pier Alberto Bertazzi, Pietro Apostoli, Lifang Hou, Andrea A Baccarelli

**Affiliations:** 1 Laboratory of Environmental Epigenetics, Exposure Epidemiology and Risk Program, Harvard School of Public Health, Boston, MA, USA; 2 Department of Environmental and Occupational Health, Università degli Studi di Milano and IRCCS Ca’ Granda Maggiore Policlinico Hospital, Milan, Italy; 3 Department of Statistics, University of Milano‒Bicocca, Milan, Italy; 4 Department of Experimental and Applied Medicine, Occupational Medicine and Industrial Hygiene, University of Brescia, Brescia, Italy; 5 Department of Preventive Medicine, Feinberg School of Medicine, Northwestern University, Chicago 60611, USA

**Keywords:** Environment, Exposures, DNA methylation, Repetitive elements, Subfamily

## Abstract

**Background:**

Repetitive elements take up >40% of the human genome and can change distribution through transposition, thus generating subfamilies. Repetitive element DNA methylation has associated with several diseases and environmental exposures, including exposure to airborne pollutants. No systematic analysis has yet been conducted to examine the effects of exposures across different repetitive element subfamilies. The purpose of the study is to evaluate sensitivity of DNA methylation in differentially‒evolved LINE, *Alu,* and HERV subfamilies to different types of airborne pollutants.

**Methods:**

We sampled a total of 120 male participants from three studies (20 high-, 20 low-exposure in each study) of steel workers exposed to metal-rich particulate matter (measured as PM_10_) (Study 1); gas-station attendants exposed to air benzene (Study 2); and truck drivers exposed to traffic-derived elemental carbon (Study 3). We measured methylation by bisulfite-PCR-pyrosequencing in 10 differentially‒evolved repetitive element subfamilies.

**Results:**

High-exposure groups exhibited subfamily-specific methylation differences compared to low-exposure groups: L1PA2 showed lower DNA methylation in steel workers (*P*=0.04) and gas station attendants (*P*=0.03); L1Ta showed lower DNA methylation in steel workers (*P*=0.02); *Alu*Yb8 showed higher DNA methylation in truck drivers (*P*=0.05). Within each study, dose–response analyses showed subfamily-specific correlations of methylation with exposure levels. Interaction models showed that the effects of the exposures on DNA methylation were dependent on the subfamily evolutionary age, with stronger effects on older LINEs from PM_10_ (p‒interaction=0.003) and benzene (p‒interaction=0.04), and on younger *Alu*s from PM_10_ (p-interaction=0.02).

**Conclusions:**

The evolutionary age of repetitive element subfamilies determines differential susceptibility of DNA methylation to airborne pollutants.

## Background

Approximately half of the human genome is made of repetitive elements [1]. Repetitive elements, particularly L1s, are DNA sequences that only encode for proteins instrumental to their replication and insertion into new locations within the genome. Also often referred to as “junk DNA” [2] or “jumping genes” [3], repetitive elements have been active in mammalian genomes for over 100 million years and are therefore a source of evolution and genome structure organization. Repetitive elements are believed to have reshaped the human genome through continuous jumping to remote genomic locations and through the activation of alternative transcription of nearby genes [4-7]. *De novo* retrotransposition insertions occurring in the germ line are inherited through generations. However, many newly inserted repetitive elements have been observed in somatic tissues and, albeit non heritable, have been linked with human disease and cancers [8,9].

Among repetitive elements, the best studied families are the long interspersed nuclear elements (LINE-1 or L1), short interspersed nuclear element family (SINEs), and human endogenous retrovirus (HERV) family. *Alu* and long-terminal repeats (LTRs) are the most abundant representatives of SINEs and HERVs, respectively. All these families are classified as retroelements because of their ability to retrotranspose, i.e., copy themselves into an RNA intermediate and insert back into the genome as a new cDNA copy [10]. Among repetitive elements, only LINE-1 and *Alu* have been unequivocally shown to be still active and retrotransposition-competent in the current human genome [11]. Since the mobilization of repetitive elements has been linked to genomic instability and consequent genetic disorders, mechanisms such as DNA methylation are believed to have developed in cells to control the proliferation of retrotransposons. CpG-dinucleotide DNA methylation in repetitive elements has been proposed to contribute to repress retrotransposition activity as part of a self-defense system of the host genome [12,13]. On the other hand, decreased DNA methylation in repetitive elements has been linked with increased transcription and higher rates of retrotransposon activity *in vitro*[14,15]. In human studies, differences in DNA methylation of LINE-1, *Alu*, and HERV have been consistently demonstrated in response to stress [16] and infections [17], as well as in autoimmune diseases [18], cardiovascular diseases [19] and cancers [20,21].

Through their “copy and paste” retrotransposition activity, each repetitive element family has gradually accumulated new base substitutions, insertions or deletions referred to as “diagnostic mutations” [22], which allow for distinguishing various subfamilies within each repetitive element family. Through the analysis of these mutations and the divergence of the repetitive element consensus sequences from the original ancestral sequence, it is possible to estimate an “evolutionary age” for each subfamily [1,23]. The mutation rate depends on the CpG density; since CpG di-nucleotides are more sensitive than non-CpG sites, they tend to be eliminated through evolution by substitution to either TpG or CpA dinucleotides. These substitutions are frequent events, as the mutation rate of CpG sites is 9.2 fold faster than non-CpG changes [24,25]. In the mammalian genome, CpG dinucleotides are found to be highly represented in repetitive elements [26]. Through many cycles of substitutions, old subfamilies remain less rich in CpGs and show weakened or no retrotransposon activity, whereas young subfamilies are richer in CpGs and still transcriptionally active in the human genome [25,27].

Recent investigations have repeatedly linked DNA methylation of repetitive elements with environmental exposures. In human studies, methylation of repetitive elements in blood DNA has been correlated with a number of exposures that generate oxidative stress and inflammation [28,29], including airborne pollutants [21,30-32], metals [33-35], and persistent organopollutants [36,37]. Several investigators have proposed that these effects may contribute to generate – or at least reflect – the altered retrotransposon methylation states related with aging, autoimmune diseases (e.g., multiple sclerosis, lupus), cardiovascular disease, or cancer development and progression [38]. DNA methylation of repetitive elements has been also widely proposed as an indicator of global genomic methylation level [39]. However, current investigations have only focused on the analysis of a single common sequence for each family, which is not expected to comprehensively reflect DNA methylation patterns within specific subfamilies. Recent reports have shown that methylation of an individual common sequence is not correlated with the global content of methylation in normal tissues [7]. A recent observational study showed that CpG content is a primary predictor of changes over time in DNA methylation at individual CpG sites [40]. Because repetitive element CpG content and DNA methylation levels vary dramatically across subfamilies with different evolutionary age, repetitive element subfamilies may also have differential sensitivity to environmental exposures. However, no systematic analysis has yet been conducted to examine the effects of environmental exposures across different subfamilies of repetitive elements.

In the present work, we conducted a comprehensive investigation of DNA methylation in repetitive element subfamilies of different evolutionary ages. We examined multiple groups of participants exposed to different types of airborne pollutants (Table 1). We sampled groups with high- and low-exposure from three independent studies including steel workers and low-exposed controls to metal-rich particulate matter (Study 1); gas station attendants and low-exposed controls to traffic-derived benzene (Study 2); and truck drivers and low-exposed controls to traffic-derived Elemental Carbon (EC) in Beijing, China. We examined DNA methylation of four LINE-1 subfamilies (L1PA5, L1PA2, L1Hs, and L1Ta) [41], three *Alu* subfamilies (*Alu*Sx*, Alu*Yb8*,* and *Alu*Yd6) [42], and three HERV subfamilies (MLT1D, ERV1, and ERV9) [43] selected within each family to represent different evolutionary ages ranging from old to young elements. We showed that the effects of the exposures on DNA methylation are dependent on the subfamily evolutionary age. To the best of our knowledge, this is the first report showing that the evolutionary age of repetitive elements determines their vulnerability to environmental exposures.

**Table 1:** Characteristics and exposure levels of the study participants

				
**Study 1**^a^ Exposure to metal-rich particulate matter (PM)			**Highly-exposed steel workers (n=20)**	**Low-exposed controls (n=20)**
	PM_10_ [μg/m^3^]	Mean ± SD	203.7 ± 22.9	100.9 ± 28.9
		Range	[152.2 ; 227.9]	[73.7 ; 150.0]
	Participants’ characteristics	Age [Years], mean ± SD	42.4 ± 7.9	37.8 ± 3.0
		Ex/current smokers, n (%)	12 (60)	14 (70)
**Study 2**^b^ Exposure to air benzene			Gas station attendants (n=20)	Low-exposed controls (n=20)
	Air benzene [μg/m^3^]	Mean ± SD	78.6 ± 42.5	7.0 ± 5.5
		Range	[31.2 ; 180.1]	[4.2 ; 23.0]
	Participants’ characteristics	Age [Years], mean ± SD	39.9 ± 11.2	39.7 ± 10.4
		Ex/current smokers, n (%)	7 (35)	5 (25)
**Study 3**^c^ Exposure to traffic-derived elemental carbon			Truck drivers (n=20)	Low-exposed controls (n=20)
	Elemental carbon [μg/m^3^]	Mean ± SD	21.3 ± 4.7	13.4 ± 2.1
		Range	[16.6 ; 35.6]	[7.8 ; 16.1]
	Participants’ characteristics	Age [Years], mean ± SD	35.2 ± 5.1	33.4 ± 6.0
		Ex/current smokers, n (%)	8 (40)	6 (30)

The participants were recruited from different occupations and divided in high- and low-exposed group according to their personal levels of exposure.

^a^ Steel workers with Particulate Matter with aerodynamic diameter <=10 μm [PM_10_] >152.2 μg/m3 in the highly-exposed group; steel workers with PM_10_<150.0 μg/m3 in the low-exposed control group.

^b^ Gas station attendants in the highly-exposed group; indoor office workers in the low-exposed control group.

^c^ Truck drivers in the highly-exposed group; indoor office workers in the low-exposed control group.

## Results

### Repetitive element evolutionary age, CpG density and methylation levels

As a measure of CpG density we calculated the ratio between observed and expected CpG content (CpG_o/e_) for each subfamily (Table 2). The subfamily evolutionary age and CpG density showed opposite directions, as younger subfamilies showed higher CpG density. The subfamily evolutionary age and DNA methylation levels – estimated among all the low-exposed control groups from Study 1, Study 2, and Study 3 – also showed opposite directions, as younger subfamilies tended to have higher mean methylation, except for HERV (Table 2).

**Table 2:** Characteristics of the repetitive element subfamilies examined in the present study

**Repetitive element**		**Evolutionary age (Mya)**^a^	**CpG density (CpG**_**o/e**_**)**^b^	**Mean methylation (%)**^c^
**Family**	**Subfamily**			
	L1PA5	20.4	0.073	25.3
**LINE-1**	L1PA2	7.6	0.104	70.6
	L1Hs	5	0.155	79.5
	L1Ta	1.9	0.282	70.1
	*Alu*Sx	40	0.878	24.7
***Alu***	*Alu*Yb8	2.9	0.884	89.9
	*Alu*Yd6	2	0.95	89.9
	MLT1D	98.2	0.043	97.9
**HERV**	ERV1	24.4	0.074	24.5
	ERV9	15	0.439	52

^a^ Estimated time in ‘Million years ago’ (Mya) based on the time when the repetitive element subfamily appeared in the human genome.

^b^ Approximate ratio of the observed to the expected CpG content (CpGo/e) of the sequence. CpG observed/expected (CpGo/e). The ratio is calculated using the formula ((Num of CpG/(Num of C × Num of G)) × Total number of nucleotides in the sequence).

^c^ Mean methylation of all low-exposure control groups from Study 1, Study 2, and Study 3.

### DNA methylation of repetitive element subfamilies by exposure group

**Figure 1:**
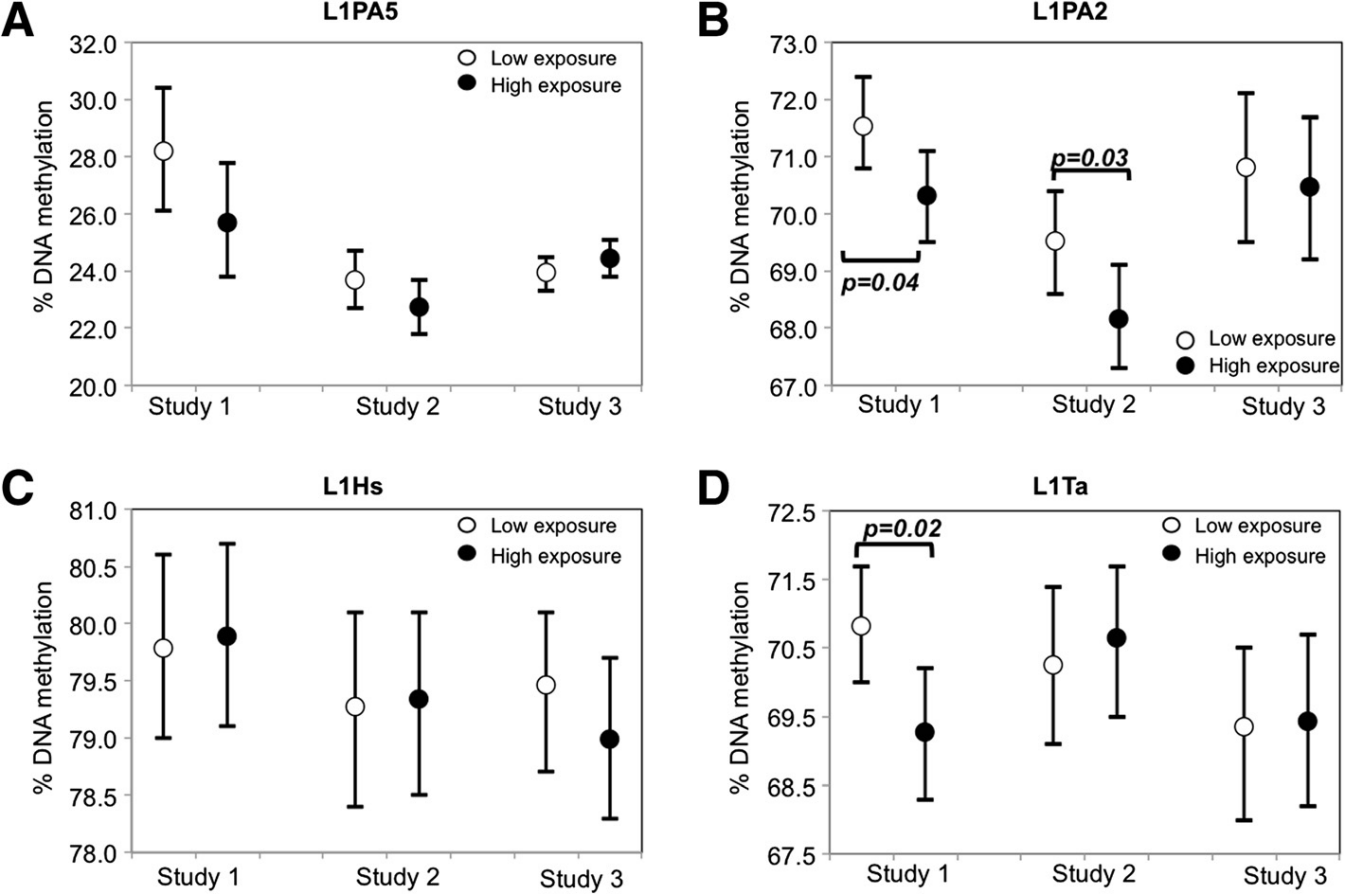
**DNA methylation differences in LINE-1 subfamilies between low and high exposure groups. **Mean DNA methylation levels and 95% confidence intervals of L1PA5 (panel **A**), L1PA2 (panel **B**), L1Hs (panel **C**), and L1Ta (panel **D**) in low and high exposure groups are shown for each of the studies. Open circles represent low exposure group (Steelworkers in low exposure job position in Study 1; indoor office workers in Study 2 and Study 3); closed circles represent high exposure group (Steel workers in high exposure job position in Study 1; gas station attendants in Study 2; and truck drivers in Study 3). Significant p-values (< 0.05) for DNA methylation differences between low and high exposure groups are shown in the figures.

**Figure 2:**
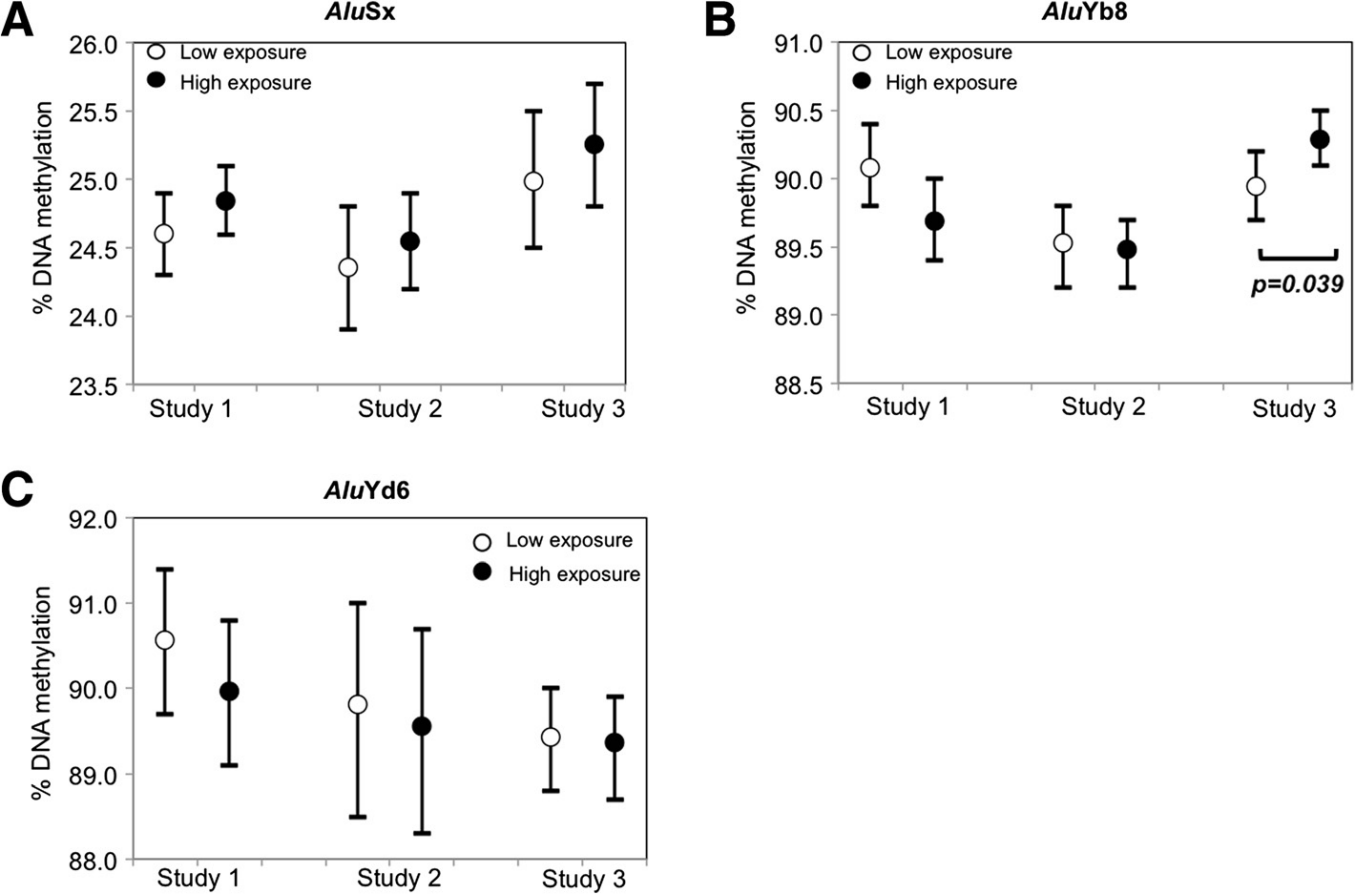
**DNA methylation differences of *Alu *subfamilies between low and high exposure group. **Mean DNA methylation levels and 95% confidence intervals of *Alu*Sx (panel **A**), *Alu*Yb8 (panel **B**), and *Alu*Yd6 (panel **C**) in low and high exposure groups are shown for each study. Open circles represent low exposure group (Steel workers in low exposure job position in Study 1; indoor office workers in Study 2 and Study 3); closed circles represent high exposure group (Steel workers in high exposure job position in Study 1; gas station attendants in Study 2; and truck drivers in Study 3). Significant p values (< 0.05) for DNA methylation differences between low and high exposure groups are shown in the figures.

We first examined DNA methylation in repetitive element subfamilies by contrasting the high- vs. low-exposure groups in analysis adjusted for age and smoking. Among LINE-1 subfamilies, L1PA2 showed significantly lower DNA methylation both in highly PM_10_-exposed steel workers in Study 1 (mean differences=-1.2%, *P*=0.04), and in gas station attendants in Study 2 (mean differences=-1.3%, *P*=0.03) relative to the respective low-exposed controls (Figure 1B and see Additional file 1: Table S1). L1Ta, the youngest of the LINE-1 showed in Study 1 significantly lower DNA methylation in steel workers with high exposure to metal-rich PM_10_ compared to the low-exposed controls (mean differences=-1.5%, *P*=0.02) (Figure 1D and see Additional file 1: Table S1). Neither L1PA5 – the oldest LINE-1 subfamily in this study – nor L1Hs, a relatively young subfamily, showed significant DNA methylation differences between low and high exposure groups in any of the three studies (Figure 1A and 1C; and see Additional file 1: Table S1). *Alu*Yb8 –a relatively younger *Alu –* showed in Study 3 significantly higher DNA methylation in truck drivers with high EC exposure compared to indoor office workers (mean difference=0.4%, *P*=0.039) (Figure 2B and see Additional file 1: Table S1). Neither *Alu*Sx – the oldest *Alu* subfamily in this study, nor *Alu*Yd6 – the youngest *Alu* subfamily in this study – showed significant DNA methylation differences between high and low-exposure groups in any of the three studies (Figure 2A and 2C; and see Additional file 1: Table S1). None of the HERV subfamilies showed significant DNA methylation differences between high and low-exposure groups in any of the three studies (see Additional file 1: Table S1).

### Dose–response relationship in the correlation of DNA methylation in repetitive element subfamilies with exposure levels

To evaluate the dose–response relationships between exposure levels and DNA methylation, we fitted regression models estimating the relative change – expressed as percent – of the ratio of methylated/unmethylated DNA associated with an increase in the exposure from the 25th to the 75th percentile, adjusted for age and smoking (Table 3). Among LINE-1 subfamilies, all the significant correlations of exposure levels with DNA methylation across studies were observed in the two oldest subfamilies, i.e., L1PA5 and L1PA2. In Study 1, DNA methylation of L1PA5 and L1PA2 was negatively correlated with the levels of exposure to metal-rich PM_10_ (τ=-15.2, *P*=0.02 and τ=-5.8, *P*=0.03, respectively). In Study 2, L1PA2 methylation showed a negative correlation with air benzene exposure (τ=-4.3, *P*=0.01). In Study 3, L1PA5 methylation showed a negative correlation with EC exposure (τ=5.6, *P*=0.01). In all the three studies, there were no or marginal correlations of methylation of *Alu*s and HERVs with the levels of exposure (Table 3).

**Table 3:** Dose–response relationship between levels of personal air pollutants exposure and DNA methylation in the participants

**Repetitive element**		**Study 1 associations with metal-rich particulate matter (PM**_**10**_**)**		**Study 2 associations with air benzene**		**Study 3 associations with traffic-derived elemental carbon**	
**Family**	**Subfamily**	**τ**^a^	**p value**	**τ**^a^	**p value**	**τ**^a^	**p value**
	L1PA5	-15.2	0.02^*^	-2.3	0.28	5.6	0.01^*^
**LINE-1**	L1PA2	-5.8	0.03^*^	-4.3	0.01^*^	-2.3	0.51
	L1Hs	1.4	0.68	1.0	0.60	-2.7	0.19
	L1Ta	-4.2	0.17	0.7	0.74	1.6	0.68
	*Alu*Sx	0.0	1.00	1.0	0.21	0.0	1.00
***Alu***	*Alu*Yb8	-3.8	0.11	-0.4	0.74	2.4	0.14
	*Alu*Yd6	-6.6	0.31	-4.1	0.37	4.0	0.29
	MLT1D	-47.6	0.16	54.6	0.13	-41.8	0.09
**HERV**	ERV1	0.4	0.78	0.6	0.56	0.0	0.99
	ERV9	1.9	0.45	1.4	0.42	0.5	0.83

^a^*τ* = (2^*β*^ - 1) * 100 represents the percent-change of the ratio methylated/unmethylated associated with an increase in the exposure from the 25th to the 75th percentile, adjusted for age and smoking.

^*^ p< 0.05.

### Evolutionary age of subfamilies and sensitivity of DNA methylation to airborne pollutants

**Figure 3:**
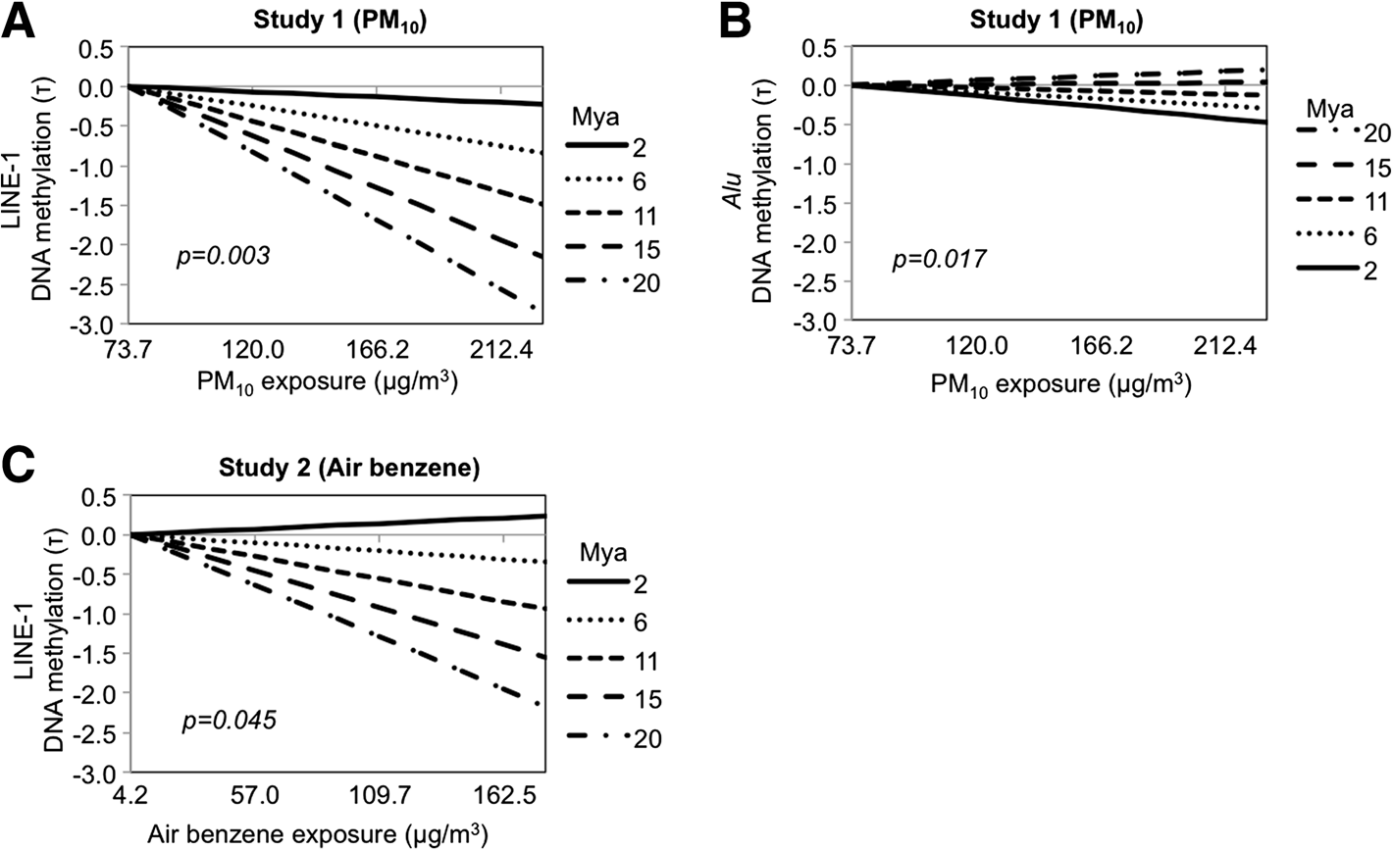
**Interaction of evolutionary ages (Mya) with air pollutant exposures in determining repetitive element DNA methylation. **Effects on DNA methylation estimated by modeling interactions of metal-rich PM_10 _exposure with ages of LINE-1 subfamilies in Study 1 (panel **A**); interactions of metal-rich PM_10 _exposure with ages of *Alu *subfamilies in Study 1 (panel **B**); interactions of airborne benzene exposure with ages of LINE-1 subfamilies in Study 2 (panel **C**). The differences in DNA methylation in each subfamily are represented using τ. The evolutionary ages shown are arbitrary numbers at regular intervals of 2, 6, 11, 15, and 20 million years ago (Mya), reflecting a plausible range and spaced intervals of evolutionary ages. The interaction p-values are shown in the figures.

To determine whether repetitive element sensitivity to environmental exposures was dependent on evolutionary age, we used an interaction analysis that modeled the correlation between environmental exposure levels and DNA methylation as a function of the evolutionary age. Figure 3 shows a graphical representation of the results for the combinations of repetitive element families and exposures that showed significant interactions with arbitrary numbers at regular intervals of 2, 6, 11, 15, and 20 million years ago (Mya), reflecting a plausible range and spaced intervals of evolutionary age, as well as for those that showed significant correlations of methylation of any of the subfamilies with the exposure levels in the section above. Complete results are shown in Additional file 1: Table S2.

In Study 1, the negative effect of PM_10_ exposure on LINE-1 methylation was stronger with increasing evolutionary age (τ for interaction=-0.6; *P*=0.003) (Figure 3A). Also in Study 1, the interaction model showed a positive significant interaction between PM_10_ exposure and evolutionary age in predicting *Alu* family methylation (τ=0.2 and *P*=0.017) (Figure 3B). However, as shown in the slopes in Figure 3, the differences of the effects of PM_10_ in Study 1 across different evolutionary ages were more pronounced for LINE-1 methylation (Figure 3A) compared to *Alu* methylation (Figure 3B). In Study 2, the negative effect of airborne benzene in LINE-1 DNA methylation was also progressively stronger with increasing evolutionary age (τ for interaction=-0.2; *P*=0.045) (Figure 3C). No interactions were found between evolutionary ages and exposure levels in determining HERV methylation (see Additional file 1: Table S2).

## Discussion

DNA methylation of repetitive elements has been extensively studied in relation to environmental exposures and human disease. Nonetheless, most if not all of the previous studies have investigated one single sequence in one or at most two repetitive element subfamilies. The present work is based on a comprehensive methylation analysis of 10 repetitive element subfamilies that were examined in three groups of participants with well-characterized exposure, including steel workers with exposure to PM_10_ in Study 1; gas station attendants exposed to air benzene in Study 2; and truck drivers exposed to EC in Study 3. We found that effects on DNA methylation of individual repetitive element subfamilies were specific to the exposure type. We selected different ages of repetitive element subfamilies – including old, intermediate, and young subfamilies – based on the time when they appeared in the human genome. We showed exposure-related effects that depended on the subfamily evolutionary age.

The different susceptibility of repetitive element methylation to environmental pollutants may be explained by the sequence variation and GC-content differences between the subfamilies. Subfamilies with older evolutionary age have lower CpG content due to higher substitution rates. As a measure of CpG density, we calculated the ratio between observed and expected CpG content (CpG_o/e_) for each subfamily (Table 2). Our data confirmed that the subfamily age was inversely correlated with DNA methylation levels in the CpG sites. Also, DNA methylation of those CpG sites was positively correlated with the ratio of CpG_o/e_. These findings show that older subfamilies have lower CpG density and are prone to have lower DNA methylation. This also supports the concept that each repetitive element family has different patterns of DNA methylation, which might reflect varying degrees of regulation and help explaining the different responses to environmental exposures.

An alternative potential explanation for the exposure-related differences in DNA methylation observed in the present study relates to the genomic position of repetitive elements in the genome. Repetitive element families show different insertional preference in the human genome; for instance, LINE-1s are frequently inserted in AT rich regions, as TTTT/A is the site to prime reverse transcription [44]. *Alu*s and HERVs are more likely inserted into GC rich regions, i.e., in regions near genes or gene-related features such as CpG islands [45]. In some instances, methylation spreads from upstream *Alus* into the nearby gene-promoter region [46]. Due to the functional relationships between repetitive elements and their surrounding regions, it is possible that differential sensitivity of the insertion regions to environmental exposures could affect DNA methylation of the inserted repetitive element. The methylation difference in this pool of repetitive elements is small (less than 2% in low versus high exposed groups) when expressed in percentage over the total number of cytosines in the considered position. Since we designed subfamily-specific assays, even a small difference in methylation levels might lead to instability of the genomic structure.

Methylation of individual sequences in the repetitive element families LINE-1, *Alu*, and HERV has been already investigated in relation to environmental exposures such as PM_10_, black carbon, and persistent organic pollutants [21,30-37,44]. However, due to the similarity of the sequences and the difficulty in designing primers for specific subfamilies, most of previous studies have analyzed only one single subfamily (i.e., L1Hs for the LINE-1 family and *Alu*Sx for the *Alu* family) and – to the best of our knowledge – no study has yet investigated multiple subfamilies. In the present study, we found significant associations of DNA methylation of specific repetitive element subfamilies in both the analysis using exposure groups (high vs. low) and in the dose–response analysis using continuous levels of exposure to metal-rich PM_10_, air benzene, or EC. However, not all the effects on DNA methylation were consistently found in both the group and dose–response analysis. For instance, L1Ta showed a significant difference in highly-exposed steel workers in Study 1, but the group analysis was not confirmed in the dose–response analysis using continuous PM_10_ levels_._ Similarly, in the *Alu* family, DNA methylation of the intermediate-age *Alu*Yb8 sequence showed a significant difference in the highly-exposed group of truck drivers in Study 3, which was not confirmed in the dose–response analysis using continuous EC levels_._ The small number of individuals in each group might explain at least part of these inconsistencies. It is worth noting that, even in the cases with discordance of statistical significance, group and dose–response analysis were concordant in showing similar directions for the exposure-related methylation differences.

Repetitive element subfamilies were inserted in the host human genome at different evolutionary ages. To provide more stable estimates of the general effects of air pollutants, as well as to elucidate the biological bases of the heterogeneity of effects within repetitive element family, we investigated whether the correlations between subfamily methylation and exposures depended on the subfamily evolutionary age. We applied arbitrary numbers at regular intervals of 2, 6, 11, 15, and 20 million years ago (Mya), reflecting a plausible range and spaced intervals of evolutionary ages. We observed that the effects of air pollutants on repetitive element methylation – particular in LINE-1 subfamilies – were significantly affected by the age of subfamilies. The interaction analysis of environmental exposure and ages of repetitive element subfamilies suggested that DNA methylation in older LINE-1 subfamilies might be more vulnerable to environmental exposure than in younger subfamilies.

Our findings are consistent with the hypothesis that exposures activate pollutant-specific biological pathways, which may in turn result in signature differences in DNA methylation in specific repetitive element subfamilies. DNA methyltransferases (DNMTs) play a fundamental role in the generation of DNA methylation by transferring methyl groups from S-adenosyl-methionine (SAM) to the C5 position of the pyrimidine ring of cytosines. DNMTs are environmentally sensitive [9] and may represent vulnerable targets in the biological process linking pollutant exposures to DNA methylation. Specific DNMT isoforms show different sensitivity to the environment, as potentially each pollutant might target one or a combination the several DNMT isoforms. DNMT isoforms also have different activity in the methylation of individual repetitive element subfamilies [47]. For instance *in vitro* studies showed that LINE-1 sequences are preferentially methylated by the DNMT3B1, DNMT3B2, and DNMTΔ3B isoforms, which however do not produce any methylation on *Alu*Yb8. Taken together, these data indicate that different susceptibility of DNMTs to environmental exposures could modify their subfamily-specific activity on DNA methylation.

The present study has a number of limitations. The small sample size of 40 participants from each of the three studies might have limited the power to detect exposure-related differences. Despite designing PCR primers on highly homologous sequence regions between subfamilies, our assays might have missed some copies of each subfamily due to the sequence variations inherent to repetitive element subfamilies. In addition, because of the general characteristics of sodium-bisulfite conversion, we could not distinguish between CpG to TpG mutation and cytosine methylation in CpG sites. Separate genomic sequencing would be necessary to identify *bona-fide* cytosine methylation in CpG sites. The selection of repetitive elements in this study was limited to representative sequences with different evolutionary ages. Future studies are needed including larger numbers of subfamilies. Nonetheless, to the best of our knowledge, the present study includes the most comprehensive selection to date of subfamilies ever examined in relation with environmental exposure.

This study has also a number of strengths that support the validity of the results. We conducted a comprehensive DNA methylation analysis of repetitive element subfamilies of different evolutionary ages within three distinct families. We used a highly quantitative bisulfite-PCR-pyrosequencing approach for DNA methylation analysis, which is the gold standard for DNA methylation analyses in short (up to 80–100 bp) sequences. Finally, we evaluated three different airborne pollutants, whose exposure was assessed through directly measured or estimated levels at the personal level. Significant differences in methylation level were found in repetitive element subfamilies that had not yet been analyzed in environmental studies. Our results suggest that previous studies that aimed at evaluating global genomic methylation by just considering specific subfamilies of L1HS and *Alu*Sx might have missed significant associations of repetitive element DNA methylation with environmental exposures. Using an augmented panel of repetitive element subfamilies might help to identify novel effects of environmental exposures.

## Conclusions

In conclusion, the present study on DNA methylation of 10 repetitive element subfamilies showed family- and subfamily-specific effects in three distinct conditions of exposures to different types of air pollutants. We also showed that sensitivity to environmental exposures is dependent on the evolutionary ages of the repetitive element subfamilies. Our results provide better understanding of the effects of the exposures on methylation of repetitive element subfamilies and may help to elucidate the role of repetitive elements in response to environmental risk factors related to human health and disease.

## Methods

### Study participants and exposure levels

We selected a total of 120 healthy individuals who participated in three previous studies of different types of airborne pollutants. From each of the three studies, we selected two groups of 20 highly-exposed individuals and 20 low-exposed controls matched by age and smoking status. In consideration of the predominance of males in the three original studies and to limit potential confounding by gender, we only sampled male participants. Table 1 shows the characteristics and the exposure levels of the three studies.

In Study 1, the participants were recruited from steel workers in Brescia, Italy [48]. In brief, we selected among workers in a steel production plant 20 steel workers with high exposure to metal-rich particles (Particulate Matter with aerodynamic diameter >10 μm [PM_10_] ≥ 152.2 μg/m^3^) and 20 office workers as controls with low exposures (PM_10_ ≤ 150.0 μg/m^3^).

In Study 2, we selected 20 gas station attendants exposed to airborne benzene in Milan, Italy as the highly-exposed group (air benzene ≥ 31.2 μg/m^3^); and 20 office workers as the low-exposed control group (air benzene ≤ 23.0 μg/m^3^) [31].

In Study 3, we selected 20 truck drivers with exposure Elemental Carbon (EC), taken as a tracer of traffic particles, in Beijing, China as the highly-exposed group (EC ≥ 16.6 μg/m^3^); and 20 office workers as the low-exposed control group (EC ≤ 16.1 μg/m^3^) [49].

Exposure assessment is described in the Additional file 1: Table S3.

### Sample preparation

Sample preparation was conducted in all studies using the same standardized procedures. Buffy coat from whole blood was collected from each participant and immediately stored at -80°C until genomic DNA was isolated. Genomic DNA from all participants within each study was isolated with the same batch of reagents in narrow time windows in order to minimize technical and operator variations. The isolated genomic DNA was stored at -80°C for future use. Details on sample collection and DNA isolation for each study have been described in previous publications [30,31,49].

### DNA methylation analysis

One μg of genomic DNA was treated using the EZ DNA Methylation Kit (Zymo Research, Orange, CA, USA) according to the manufacturer’s protocol. Final elution was performed with 30 μL M-Elution Buffer. Bisulfite-treated DNA was aliquoted and stored at -80°C until ready for use. We performed DNA methylation analyses on bisulfite-treated DNA using highly quantitative analysis based on PCR-pyrosequencing. The PCR and pyrosequencing primer sequences for L1HS, *Alu*Sx, and *Alu*Yb8 have been previously published by Yang *et al*. [39] and by Choi *et al*. [50]. We developed additional assays specific for three LINE-1 subfamilies (L1PA5, L1PA2, and L1Ta), for *Alu*Yd6 subfamily, and for three HERV subfamilies (MLT1D, ERV1, and ERV9) (see Additional file 1 for primer sequences and PCR conditions). A minimum number of 1 CpG and a maximum of 5 CpGs were evaluated in each assay. PCR amplification was performed at standard conditions using the GoTaq^®^ Hot Start Polymerase (Promega, Madison, WI).

We used a PSQ Q96 MD pyrosequencing System (QIAGEN, Valencia, CA), as previously described [30]. Each sample was tested two times for each assay to confirm reproducibility. As a quality control check to estimate bisulfite conversion efficiency, we placed duplicate genomic DNA samples on each bisulfite conversion plate to estimate the internal plate variation of bisulfite conversion and the pyrosequencing reaction. We also added universal PCR products amplified from cell-line DNA on each pyrosequencing plate to check run-to-run and plate-to-plate variation. In addition, the Pyrogram peak pattern from every sample was visually inspected to confirm the quality of the reaction.

### Characteristics of the repetitive element subfamilies

The repetitive element evolutionary age is defined based on the estimated time when each subfamily appeared in the human genome. We identified an evolutionary age (expressed in Mya [million years ago]) for each repetitive element subfamily based on information available in previous literature [41-43,51,52] (Table 2). As a measure of CpG density we calculated the ratio between observed and expected CpG content (CpG_o/e_) for each subfamily. The CpG observed/expected (CpG _o/e_) ratio is calculated by formula; ((Num of CpG/(Num of C × Num of G)) × Total number of nucleotides in the sequence) [53] (Table 2).

### Statistical analysis

We used mixed-effect regression models to evaluate the effects of the exposures on DNA methylation levels of each subfamily, as previously reported [54]. This approach yields a global estimate of the effect on multiple CpGs within each subfamily sequence by modeling correlated data in adjacent CpG sites within each sequence, as well as the measures from duplicate pyrosequencing runs. Mixed-effect models have the advantage over standard methods of using the entirety of the information in the data, thus maximizing statistical power by distinguishing different sources of variance. See Additional file 1 for the detailed description of the models used.

## Abbreviations

CpGo/e: Observed and expected CpG content; DNMT: DNA methyltransferases; EC: Elemental carbon; HERV: Human endogenous retrovirus; LINE-1 or L1: Long interspersed nuclear elements; LTR: Long-terminal repeats; PCR: Polymerase chain reaction; PM: Particulate matter; PSQ: Pyrosequencing; SAM: S-adenosyl-methionine; SINE: Short interspersed nuclear element family.

## Competing interests

The authors declare that they have no competing interests.

## Authors’ contributions

HMB, VM and AAB designed the experiment. TP analyzed the data. HMB and VM performed the experiments. PAB, PA, LF, and AAB contributed participant’s materials. HMB, VM and AAB wrote the manuscript and AAB oversaw the research. All authors have read and approved the final manuscript.

## Supplementary Material

Additional file 1: Table S1 DNA methylation in the three populations investigated by high and low exposure. **Table S2. **Interaction of evolutionary ages (Millions year ago, Mya) with airborne pollutant exposures in determining repetitive element DNA methylation. Selected results from the same analyses are reported in graphical form in Figure 3. **Table S3.** Primer sequences and PCR conditions. Click here for file
