# Supplementary material for: Evolutionary age of repetitive element subfamilies and sensitivity of DNA methylation to airborne pollutants
Source: Part Fibre Toxicol. 2013 Jul 15;10:28. doi: 10.1186/1743-8977-10-28 (PMC3717285; doi:10.1186/1743-8977-10-28)
Supplement: Additional file 1: Table S1 — DNA methylation in the three populations investigated by high and low exposure. Table S2. Interaction of evolutionary ages (Millions year ago, Mya) with airborne pollutant exposures in determining repetitive element DNA methylation. Selected results from the same analyses are reported in graphical form in Figure 3. Table S3. Primer sequences and PCR conditions. [file 1743-8977-10-28-S1.pdf]

Evolutionary Age of Repetitive Element Subfamilies and Sensitivity of DNA Methylation to Airborne Pollutants

Hyang-Min Byun[1*†], Valeria Motta[1,2*], Tommaso Panni[3], Pier Alberto Bertazzi[2], Pietro Apostoli[4], Lifang Hou[5], Andrea A. Baccarelli[1]

Table of Contents

Exposure assessment……………………………………………………………………….S3

Statistical models……………………………………………………………………….....S3

Table S1…………………………………………………………………………………….S6

Table S2…………………………………………………………………………………….S7

Table S3…………………………………………………………………………………….S8

References………………………………………………………………………………….S9

Exposure assessment

In Study 1, PM was measured using a GRIMM 1100 light-scattering dust analyzer (Grimm Technologies, Inc. Douglasville, GA, USA). Measures of airborne PM mass and PM metal components were obtained from 11 work areas of the steel production facility in order to estimate individual exposures.

In Study 2, the participants wore a passive sampler (stainless steel tube, internal diameter of 9 mm, length of 90 mm) containing Chromosorb 106, near the breathing zone during the work shift. Air benzene level in the passive sampler was measured by thermal desorption followed by gas chromatography/flame ionization detector analysis.

In Study 3, we measured personal EC levels using gravimetric samplers worn near the breathing zone by the study participants during the eight hours of work. Each sampler setup included an Apex pump (Casella Inc., Bedford, UK), a Triplex Sharp-Cut Cyclone (BGI Inc., Waltham, Massachusetts), and a 37-mm Teflon filter placed on top of a drain disc and inside a metal filter holder. The filters were kept under atmosphere-controlled conditions before and after sampling and were weighed with a microbalance (Mettler-Toledo Inc., Columbus, Ohio, USA).

Statistical models

We used the following model:

[1] $$Y_{ijk} = \beta_0 + \beta_1 exposure + \beta_2 X_2 + \cdots + \beta_p X_p + \upsilon_{0i} + \upsilon_{1ij} position + \epsilon_{ijk}$$

where Y_{ijk} represents the methylation level for the *i*-th subject, the *j*-th position and the *k*-th duplicate run (*i=1,…,40*; *j=1,…,m,* where m varies depending on the total number of CpG sites measured in the sequence; and *k=1,2*). υ_{0i} *and* υ_{1ij} are the random intercept for subject and

random slope for CpG position, respectively. β_0 is the overall intercept and β_1 is the fixed effect which expresses the association between exposure and DNA methylation. $X_2 \ldots X_p$ and $\beta_2 \ldots \beta_p$ represent covariates and their regression coefficients; ϵ_{ijk} is the residual term error. Age and smoking were considered *a priori* as possible confounders and therefore included as covariates in all the models of the analysis.

We first fitted a set of models in which DNA methylation was regressed over dichotomous exposure variables (high-exposure vs. low-exposure control groups). In a second set of models, we evaluated dose-response relationships by regressing DNA methylation over continuous exposure-level variables. To increase goodness-of-fit, all continuous exposure variables were log-transformed. As suggested by Du et al. (Du et al. 2010), we transformed the original methylation measures (bounded between 0 and 100%) in M-values (ranging between -∞ and +∞) using the following conversion: $\log_2$[*meth (%5 mC)*/(100 - *meth (%5 mC)*)] in all the models. M-values have been shown to improve homoscedasticity of methylation data and allow for more robust statistical estimates (Du et al. 2010). However, the M-value does not have an intuitive biological meaning and the corresponding model parameters do not have a straightforward interpretation. Therefore, we calculated τ as a transformation of the regression coefficients β using the formula $\tau = (2^{\beta} - 1) * 100)$, which represents the percent-change of the ratio methylated/unmethylated associated with the exposure. For continuous exposure variables, τ was scaled to represent the percent change associated with an increase in exposure from the 25th to the 75th percentile. We checked regression assumptions by performing diagnostic tests for each model, including the Shapiro-Wilk test for normality of residuals and White test for variance homogeneity of residuals.

We also used mixed-effect regression models to determine whether the correlation between DNA methylation and exposures within each repetitive element family varied as a function of the evolutionary age of the subfamilies. The corresponding model was:

$$[2]\ Y_{ijkl} = \beta_0 + \beta_1 exposure + \beta_2 subfamily\_age + \beta_3 exposure * subfamily\_age + \beta_4 X_4 + \cdots + \beta_p X_p + \upsilon_{0jk} subfamily * position + \upsilon_{1i} sample + \epsilon_{ijkl}$$

where Y_{ijkl} represents the methylation level for the i-th subject, the j-th subfamily, the k-th position and the l-th run (*i=1,…,40*; *j=1,…,m*; where m varies depending on the number of subfamilies evaluated in the family; *k=1,…,n,* where n varies depending on the total number of CpG sites measured in the sequence; and *l=1,2*). The random part of the model is composed by the slopes, υ_{0jk} and υ_{1i}, for the interaction between position and subfamily and for subject, respectively. The interaction slope was used to model the existence in the data of positions in common for all the subfamilies. Hence, by using the same labels for the positions in the same common sequences – even if belonging to different subfamilies – and rescaling the others accordingly, the interaction can describe unambiguously to which position and subfamily the measure refers. Finally, β_0 is the overall intercept; β_1 represents the fixed effect for the exposure, β_2 for the age of the subfamily; β_3 expresses the interaction between exposure and evolutionary age; $X_4 \ldots X_p$ and $\beta_4 \ldots \beta_p$ are the covariates and their regression coefficients; and ϵ_{ijkl} is the residual term error.

A two-sided $P<0.05$ was considered statistically significant. All statistical analyses were performed in SAS (version 9.2; SAS Institute Inc., Cary, NC, USA). We used the PROC MIXED procedure to run the mixed-effect models.

Table S1. DNA methylation in the three populations investigated by high and low exposure.

Repetitive Element		Study 1 Exposure to PM_{10} Brescia, Italy					Study 2 Exposure to Air Benzene Milan, Italy					Study 3 Exposure to Elemental Carbon Beijing, China				
		Steel workers in **low exposure** job position (n=20)		Steel workers in **high exposure** job position (n=20)		p value	**Low-exposed controls** (n=20)		**Gas station attendants** (n=20)		p value	**Low-exposed controls** (n=20)		**Truck drivers** (n=20)		p value
	Subtype	Mean	95% CI	Mean	95% CI		Mean	95% CI	Mean	95% CI		Mean	95% CI	Mean	95% CI	
**LINE-1**	L1PA5	28.2	(26.1;30.4)	25.7	(23.8;27.8)	0.10	23.7	(22.7;24.7)	22.7	(21.8;23.7)	0.14	24.0	(23.3;24.5)	24.4	(23.8;25.1)	0.28
	L1PA2	71.5	(70.8;72.4)	70.3	(69.5;71.1)	**0.04***	69.5	(68.6;70.4)	68.2	(67.3;69.1)	**0.03***	70.8	(69.5;72.1)	70.5	(69.2;71.7)	0.70
	L1Hs	79.8	(79.0;80.6)	79.9	(79.1;80.7)	0.85	79.3	(78.4;80.1)	79.3	(78.5;80.1)	0.90	79.5	(78.7;80.1)	79.0	(78.3;79.7)	0.30
	L1Ta	70.8	(70.0;71.7)	69.3	(68.3;70.2)	**0.02***	70.3	(69.1;71.4)	70.7	(69.5;71.7)	0.59	69.4	(68.0;70.5)	69.4	(68.2;70.7)	0.92
Alu	*Alu*Sx	24.6	(24.3;24.9)	24.8	(24.6;25.1)	0.22	24.4	(23.9;24.8)	24.5	(24.2;24.9)	0.48	25.0	(24.5;25.5)	25.3	(24.8;25.7)	0.34
	*Alu*Yb8	90.1	(89.8;90.4)	89.7	(89.4;90.0)	0.07	89.5	(89.2;89.8)	89.5	(89.2;89.7)	0.80	89.9	(89.7;90.2)	90.3	(90.1;90.5)	**0.039***
	*Alu*Yd6	90.6	(89.7;91.4)	90.0	(89.1;90.8)	0.33	89.8	(88.5;91.0)	89.6	(88.3;90.7)	0.75	89.4	(88.8;90.0)	89.4	(88.7;89.9)	0.87
**HERV**	MLT1D	98.2	(96.7;99.1)	96.2	(93.1;97.9)	0.08	97.2	(94.2;98.7)	98.7	(97.5;99.4)	0.12	98.1	(96.8;98.9)	96.8	(94.8;98.0)	0.15
	ERV1	24.4	(23.9;24.8)	24.5	(24.1;24.9)	0.63	24.1	(23.7;24.7)	24.4	(23.9;24.9)	0.38	25.0	(24.5;25.3)	25.0	(24.5;25.3)	0.90
	ERV9	53.2	(52.2;54.1)	53.6	(52.8;54.5)	0.45	53.2	(52.1;54.3)	53.3	(52.2;54.3)	0.82	49.8	(48.8;50.9)	49.8	(48.8;50.9)	0.94

The participants were divided in high- and low-exposed control group according to their exposure levels.

* p values < 0.05

Table S2. Interaction of evolutionary ages (Millions year ago, Mya) with airborne pollutant exposures in determining repetitive element DNA methylation. Selected results from the same analyses are reported in graphical form in Figure 3.

Repetitive Element	Effect	Study 1 Exposure to PM_{10} Brescia, Italy		Study 2 Exposure to Air Benzene Milan, Italy		Study 3 Exposure to Elemental Carbon Beijing, China	
		τ^a	p value	τ^a	p value	τ^a	p value
LINE-1	Exposure	0.1	0.964	1.0	0.505	-2.8	0.271
	Age	-100.0	0.044	-99.6	0.017	-100.0	0.024
	Exposure * Age	-0.6	**0.003***	-0.2	**0.045***	0.2	0.432
Alu	Exposure	-4.9	0.034	-1.8	0.233	3.1	0.043
	Age	-100.0	<0.001	-99.0	<0.001	-100.0	<0.001
	Exposure * Age	0.2	**0.017***	0.1	0.140	-0.1	0.247
HERV	Exposure	76.3	0.491	17.7	0.674	-3.8	0.954
	Age	-100.0	<0.001	-100.0	<0.001	-100.0	<0.001
	Exposure * Age	-1.2	0.266	0.3	0.582	-0.1	0.905

[a] $\tau = (2^{\beta} - 1) * 100$ represents the percent-change of the ratio methylated/unmethylated associated with an increase from the 25th to the 75th percentile of the exposure level, adjusted for age and smoking. * p values < 0.05

Table S3. Primer sequences and PCR conditions.

Family	Assay	Forward Primer (5' to 3')	Reverse Primer (3' to 5')	Sequencing Primer (5' to 3')	Annealing Temperature (°C)
**LINE-1**	L1PA5	TTAGTTAAGGGAAGAGGGGATAAA	Biotin)ATAAACATAAAACCCT CTAAACCAAACA	TTAGTTAAGGGAAGA	40
	L1PA2	TTAGATAGTGGGYGTAGGTTAGTGGGT	Biotin)CCTCCRAACCAAATAT AAAATATAATCT	GAGTTAAAGAAAGGG	55
	L1Hs	TTTTGAGTTAGGTGTGGGATATA	Biotin)AAAATCAAAAAATTCC CTTTC	AGTTAGGTGTGGGATA TAGT	56.3
	L1Ta	GGGTTAGGGAGTTTTTTTTT	Biotin)CTCTAAACCAAATATA AAATATA	GGGTTAGGGAGTTTTT TTTT	55
Alu	*Alu*Sx	Biotin)TTTTTATTAAAAATATAAAAATT	CCCAAACTAAAATACAATAA	AATAACTAAAATTACA AAC	50
	*Alu*Yb8	Biotin)AGATTATTTTGGTTAATAAG	AACTACYAACTACAATAAC	AATAACTAAAACTACA AAC	53.9
	*Alu*Yd6	Biotin)GAGATTAYGGTGAAATTT	CCCAAACAAAAATACTATAA	AATAACTAAAACTACA AAC	53.9
**HERV**	MLT1D	TATTAGGAATTGAAAGAGGGAAAGA	Biotin)TCAAAACCACTATAAA AATTACCACAA	TTTAGAGGAAGGATA	55
	ERV1	TTTGTATGGAAGGAGAAATGGTTAG	Biotin)ATACCTCTTCCCCAAAT TTCTTTAT	TTTGTATGGAAGGAGA AATGGTTAG	55
	ERV9	TGTTATTGTTTATTTTTTRGGTTTA	Biotin)TCTTCCTTCTAATAAAT TCATAATCTC	TTATTTTTGAAGTTA	55

References

Du P, Zhang X, Huang CC, Jafari N, Kibbe WA, Hou L, et al. 2010. Comparison of Beta-value and M-value methods for quantifying methylation levels by microarray analysis. BMC Bioinformatics 11: 587.
